# CRISPR/Cas12a-Based Diagnostic Platform Accurately Detects *Nocardia farcinica* Targeting a Novel Species-Specific Gene

**DOI:** 10.3389/fcimb.2022.884411

**Published:** 2022-05-27

**Authors:** Xiaotong Qiu, Shuai Xu, Xueping Liu, Hongtao Ren, Lichao Han, Zhenjun Li

**Affiliations:** ^1^State Key Laboratory for Infectious Disease Prevention and Control, National Institute for Communicable Disease Control and Prevention, Chinese Center for Disease Control and Prevention, Beijing, China; ^2^School of Laboratory Medicine and Life Sciences, Wenzhou Medical University, Wenzhou, China; ^3^Xingtai People’s Hospital, Hebei Medical University, Xingtai, China

**Keywords:** CRISPR, Cas12a, CRISPR-CLA, *pbr1*, *Nocardia farcinica*, accurate diagnosis, nucleic acid detection

## Abstract

Under the COVID-19 pandemic background, nucleic acid detection has become the gold standard to rapidly diagnose the infectious disease. A rapid, low cost, reliable nucleic acid detection platform will be the key to control next potential pandemic. In this study, a nucleic acid detection platform, which combined CRISPR/Cas12a-based detection with loop-mediated isothermal amplification (LAMP), was developed and termed CRISPR-CLA. In the CRISPR-CLA system, LAMP preamplification was employed, and CRISPR/Cas12a-based detection was used to monitor the preamplicons. The forward inner primer (FIP) was engineered with a protospacer adjacent motif (PAM) site TTTA of Cas12a effector at the linker region; thus, the CRISPR-CLA platform can detect any sequence as long as the primer design meets the requirement of LAMP. To demonstrate the validity of the CRISPR-CLA system, it was applied for the molecular diagnosis of nocardiosis caused by *Nocardia farcinica* (*N. farcinica*). A highly conserved and species-specific gene *pbr1* of *N. farcinica*, which was first reported in this study, was used as the target of detection. A set of LAMP primers targeting a fragment of *pbr1* of the *N. farcinica* reference strain IFM 10152 was designed according to the principle of CRISPR-CLA. Three CRISPR RNAs (crRNAs) with different lengths were designed, and the most efficient crRNA was screened out. Additionally, three single-strand DNA (ssDNA) probes were tested to further optimize the detection system. As a result, the *N. farcinica* CRISPR-CLA assay was established, and the whole detection process, including DNA extraction (20 min), LAMP preamplification (70°C, 40 min), and CRISPR/Cas12a-mediated detection (37°C, 8 min), can be completed within 70 min. A fluorescence reader (for fluorescence CRISPR-CLA) or a lateral flow biosensor (for lateral-flow CRISPR-CLA) can be the media of the result readout. Up to 132 strains were used to examine the specificity of *N. farcinica* CRISPR-CLA assay, and no cross-reaction was observed with non-*N. farcinica* templates. The limit of detection (LoD) of the *N. farcinica* CRISPR-CLA assay was 100 fg double-strand DNA per reaction. *N. farcinica* was detected accurately in 41 sputum specimens using the *N. farcinica* CRISPR-CLA assay, which showed higher specificity than a real-time qPCR method. Hence, the *N. farcinica* CRISPR-CLA assay is a rapid, economic and accurate method to diagnose *N. farcinica* infection.

## 1 Introduction

*Nocardia* spp., aerobic, partially acid-fast, branched gram-positive bacilli, are considered opportunistic pathogens. *Nocardia* are commonly found in patients with acute or chronic lung infections and suppurative diseases, and are long-term neglected in the clinic. *Nocardia farcinica* (*N. farcinica*) is a species of genus *Nocardia* and is one of the most common pathogens causing lung nocardiosis (Wang et al., 2022). The identification of *Nocardia* at the species level is significant because different species of *Nocardia* exhibit different drug resistance spectra (Brown-Elliott et al., 2006; Conville et al., 2018; Margalit et al., 2021). In addition, *N. farcinica* shares some phenotypic similarities with *Gordonia*, *Rhodococcus* and rapidly growing *Mycobacterium*, which may result in misidentification and misdiagnosis (Brown et al., 2004). For many patients with nocardiosis, misdiagnosis brings about delay of the illness and/or incorrect therapy, resulting in high mortality (Margalit et al., 2021). Culture of *N. farcinica* usually requires 2 to 7 days, which makes it difficult to use culture-based identification methods, such as MALDI-TOF MS. Therefore, a simple, rapid and accurate diagnostic method for *N. farcinica* analysis is urgent.

In 2004, June M. Brown et al. developed a PCR assay targeting a 314 bp species-specific DNA fragment to identify *N. farcinica* clinical isolates (Brown et al., 2004). Similarly, in 2007, Taichi et al. reported a PCR primer targeting a part of a putative gene *nfa* 29510 and a non-ORF gene of the bacterium to identify *N. farcinica* (Hasegawa et al., 2007). Although these assays exhibited good specificity in *N. farcinica* identification, PCR amplification and agarose gel electrophoresis (AGE) require specific instruments and thus their field application are limited.

Loop-mediated isothermal amplification (LAMP), which was developed by Notomi et al. in 2000, is a specific, efficient, and rapid method that allows amplification at a single temperature within one hour. A set of LAMP primers targeting eight regions of the target sequence consisted of two inner primers FIP and BIP, two outer primers F3 and B3 and two loop primers LF and LB (Notomi et al., 2000). LAMP provides more possibilities for rapid diagnosis in the field. However, false positive results may occur in LAMP due to cross-contamination, nonspecific amplification, or primer dimerization.

Recently, clustered regularly interspaced short palindromic repeats (CRISPR)/CRISPR-associated (CRISPR/Cas) systems have opened the door to new biotechnological applications, such as genome editing and transcription regulation. In particular, many nucleic acid detection platforms have been developed with the help of Cas effectors (e.g., Cas12a, Cas12b, Cas13a and Cas14), which possess both *cis* and *trans* cleavage activity (Li et al., 2018a). For example, Cas12a-based DNA Endonuclease Targeted CRISPR *Trans* Reporter (DETECTR) and one-Hour Low-cost Multipurpose highly Efficient System (HOLMES) have been used for the detection of human papillomavirus (HPV) and SNPs (Chen et al., 2018; Li et al., 2018b). Similarly, Cas13a-based Specific High Enzymatic Reporter UnLOCKing (SHERLOCK) and Cas14-based DETECTR have been used for pathogen detection with single-base mismatch specificity and attomolar sensitivity (Gootenberg et al., 2017; Harrington et al., 2018). A suitable amplification step is usually needed to improve the sensitivity of CRISPR-based detection. Therefore, LAMP is considered an alternative amplification method to aid CRISPR-based detection to achieve accurate diagnosis.

**Figure 1:**
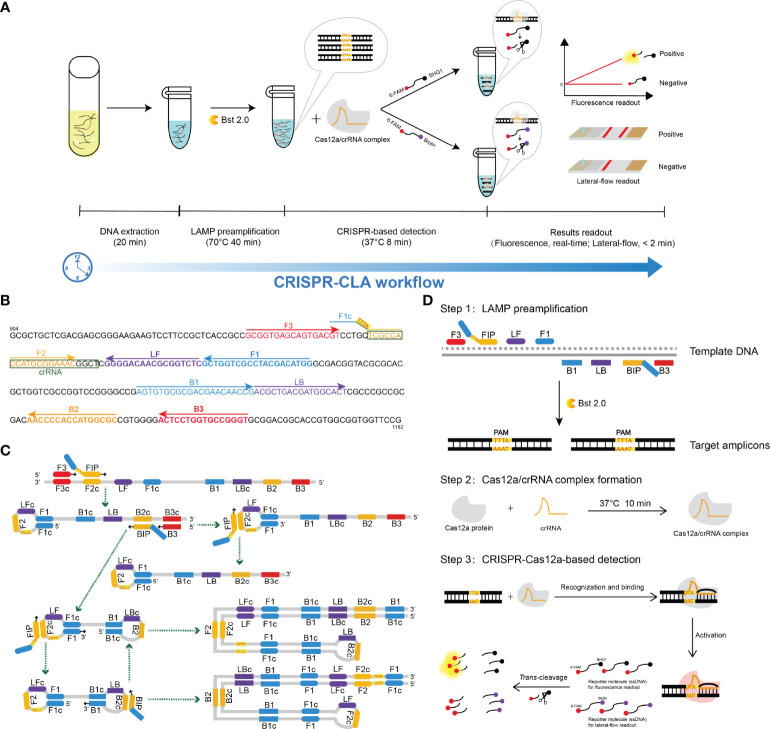
Schematic illustration of the workflow and the reaction principle of the CRISPR-CLA assay. **(A)** CRISPR-CLA workflow. The whole reaction of the CRISPR-CLA assay can be completed within 70 min. **(B)** Primers and crRNA design of the *N. farcinica* CRISPR-CLA assay. The *pbr1* gene of *N. farcinica* IFM 10152 was used to design LAMP primers. The nucleotide sequence of the sense strand of part of the *pbr1* gene (from 904 to 1182 bp) is shown. Right-pointing arrows and left-pointing arrows indicate sense and complementary sequences that were used, respectively. The inserted PAM site (TTTA) is highlighted in red and on a yellow background. The crRNA was boxed. **(C)** Principle of the PAM site inserted by LAMP preamplification. The amplification products are indicated by the corresponding green arrows. **(D)** Principle of the CRISPR-CLA system. Step one: the PAM site was introduced into the target amplicons by the modified FIP, with the target amplicons exponentially increased by LAMP. Step two: the Cas12a/crRNA complex was stably formed. Step three: Cas12a was activated after the target amplicons recognized and bound with the Cas12a/crRNA complex, and the single-strand DNA (ssDNA) reporter molecules were cleaved by activated Cas12a.

In this study, we developed a CRISPR/Cas12a-mediated nucleic acid detection platform, named CRISPR-CLA, which coupled CRISPR/Cas12a-based nucleic acid detection with LAMP and applied CRISPR-CLA to detect *N. farcinica* accurately using a novel species-specific gene, named the *pbr1* gene. Here, we expounded the basal principle of the CRISPR-CLA assay (**Figure 1**) and proved its feasibility in the diagnostic of nocardiosis caused by *N. farcinica* using pure cultures of strains and clinical specimens.

## 2 Materials and Methods

### 2.1 Reagents and Instruments

Wizard^®^ genomic DNA purification kits (A1125, Promega, USA) were purchased and used for DNA extraction. 2× Premix Ex Taq (Probe qPCR) (RR390A, Takara, China) was purchased and used for real-time qPCR detection. 10× NEBuffer 2.1 (B7202S, New England Biolabs, USA) and EnGen^®^ Lba Cas12a (Cpf1) (M0653T, New England Biolabs, USA) were purchased and used for CRISPR-based detection. DNA isothermal amplification kits (HT0600, HuiDeXin, China) were purchased and used for LAMP preamplification. A microspectrophotometer (Nanodrop ND-1000, Thermo Scientific, China) was used for DNA quantification. An isothermal metal bath (88870004, Thermo Scientific, China) was used for the preparation of the Cas12a/crRNA complex. A real-time fluorescence qPCR instrument (QuantStudio 6 Flex, Applied Biosystems, USA) was used as the fluorescence reader in this study.

### 2.2 Primers and crRNA Design

The *pbr1* gene (GenBank accession: AP006618.1), the product of which is a PQQ-binding-like beta-propeller repeat protein (GenBank accession: WP_011210687.1), was selected as the target gene because it is highly conserved and specific in *N. farcinica* (**Figure S1**). A set of LAMP primers targeting the *pbr1* gene was designed online using Primer Explorer software (version 5) (http://primerexplorer.jp/e/) based on the principle of LAMP (**Figure 1B** and **Table S1**). The specificity of the primer set was determined by nucleotide BLAST, and the secondary structure and dimer were examined by Oligo Analyzer version 3.1 (http://eu.idtdna.com/calc/analyzer). Three CRISPR RNAs (crRNAs) of different lengths were designed according to the LAMP site. The LAMP primers were synthesized and purified by Sangon Biotech. Co., Ltd. (Shanghai, China) at HPLC purification grade, and crRNA was synthesized by TianYi-Huiyan Biotech. Co., Ltd. (Beijing, China) at HPLC purification grade. The sequences of primers and crRNAs are shown in **Table S1**.

### 2.3 Real-Time qPCR Detection

A real-time qPCR assay was performed using the primers and the probe described in a previous study (Bittar et al., 2010). The 20 μL qPCR reaction system contained 10 μL of 2× Premix Ex Taq (Probe qPCR), 0.2 μM forward primer (5’-ACCGATCCGCCGTCAAATC-3’), 0.2 μM reverse primer (5’-TCGGTCGTCCGGTGTGGA-3’), 0.4 μM TaqMan probe (5’-FAM-CACATACCCCAACGCCAGCTGA-BHQ1-3’), 1 μL of template DNA and DNase/RNase-free deionized water (DW) to 20 μL. Cycling was in a QuantStudio 6 Flex instrument as follows: predenaturation at 95°C for 30 s; 40 cycles of denaturation at 95°C for 5 s, annealing and extension at 60°C for 30 s.

### 2.4 CRISPR-CLA Assay

The CRISPR-CLA assay involved two steps (**Figure 1D**). First, LAMP technology was employed to amplify the target sequence exponentially. The twenty-five microliter LAMP reaction system contained 12.5 μL of 2× isothermal amplification buffer (BF), 1 μL of *Bst* 2.0, 0.4 μM F3 and B3, 0.8 μM LF and LB, 1.6 μM FIP and BIP, 1 μL of template DNA and DW to 25 μL. In the negative control reaction, the template DNA was replaced by DW in the same volume. The CRISPR-Cas12a/crRNA complex was prepared in advance as described in a previous study (Qiu et al., 2022) and was stored at 4°C before use. The storage time of the complex did not exceed 18 hours. Second, the CRISPR/Cas12a *trans*-cleavage assay was performed in a 100 μL mixture containing 50 μL of 2× NEBuffer 2.1, 2.5 μL of single-strand DNA (ssDNA) reporter molecule (10 μM, 6-FAM/BHQ1 labeled ssDNA for fluorescence readout or 6-FAM/Biotin labeled for lateral flow biosensor (LFB) readout), 18 μL of CRISPR-Cas12a/crRNA complex, 27.5 μL of deionized water, and 2 μL of LAMP products or deionized water (blank control). The CRISPR/Cas12a-based reaction was conducted at 37°C for 8 min.

### 2.5 CRISPR-CLA Lateral Flow Biosensor Readout

**Figure 2:**
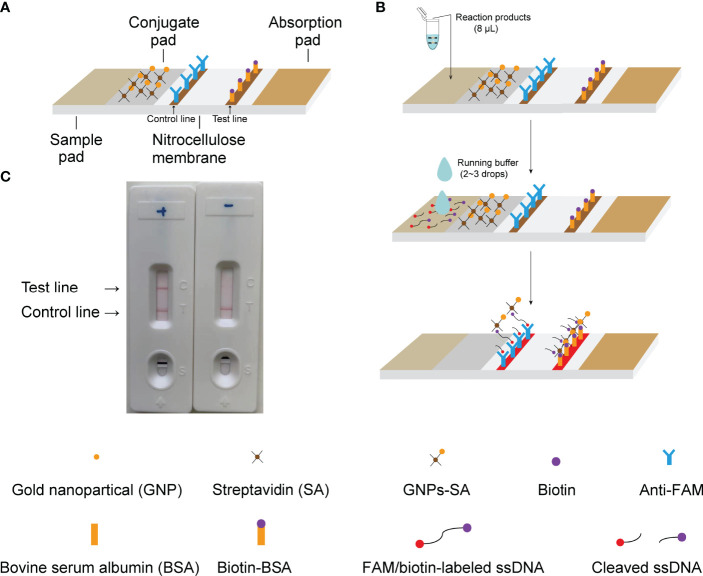
Principle of results readout of CRISPR-CLA assay by LFB. **(A)** Construction of LFB. **(B)** Principle of LFB for visualization of CRISPR-CLA products. **(C)** Practical application of LFB. Both the control line and the test line showed red bands in the positive result, and only the control line showed a red band in the negative result.

A portable LFB (**Figure 2**) was able to read out the result of CRISPR-CLA conveniently and visually. The LFB was designed according to a previous report (Zhu et al., 2021). In short, the LFB was composed of a sample pad, a conjugate pad, a nitrocellulose membrane, and an absorption pad. Gold nanoparticles-streptavidin (GNPs-SA) were laid on the conjugate pad as the indicator reagent. Rabbit anti-fluorescein antibody (anti-FAM) and biotinylated bovine serum albumin (Biotin-BSA) were distributed onto the nitrocellulose membrane as the test line and the control line, respectively.

An aliquot (8 μL) of CRISPR-CLA reaction products was loaded onto the sample pad of the LFB. Next, 2 to 3 drops (approximately 50 μL) of running buffer were added to the sample pad to flow the reagents on the biosensor. The detection results of the CRISPR-CLA assay were visible by the naked eye within two minutes (red bands on the nitrocellulose membrane, **Figure 2C**).

### 2.6 Specificity and Sensitivity of *N. farcinica* CRISPR-CLA Assay

In total, 82 N*. farcinica* strains and 50 non-*N. farcinica* isolates were collected by Biosafety Branch, National Institute for Communicable Disease Control and Prevention, Chinese Center for Disease Control and Prevention to test the specificity of the *N. farcinica* CRISPR-CLA assay (**Table S2**).

Double-strand DNA (dsDNA) of the *N. farcinica* reference strain IFM 10152 was extracted and quantified by a Nanodrop ND-1000. The dsDNA was serially diluted from 1 ng/μL to 1 fg/μL at 10-fold intervals using DW for the sensitivity test of the *N. farcinica* CRISPR-CLA assay and the real-time qPCR detection. One microliter of DNA was used as the template for each reaction. Real-time fluorescence CRISPR-CLA and lateral flow CRISPR-CLA were conducted independently to test the limit of detection (LoD) of the *N. farcinica* CRISPR-CLA assay.

All the isolates used in this study were stored in 20% (w/v) glycerol broth at −70°C until DNA extraction. Genomic DNA was extracted from cultured strains using the Wizard^®^ genomic DNA purification kit according to the technical manual.

### 2.7 Feasibility of the *N. farcinica* CRISPR-CLA Assay for Clinical Samples

In total, 41 human sputum specimens, including twenty simulated *N. farcinica*-positive sputa (S1-S20), one *N. farcinica*-positive sample from a nocardiosis patient (S21), and twenty *N. farcinica*-negative sputum samples (N1-N20) were used to validate *N. farcinica* CRISPR-CLA assay in clinical samples. One hundred microliters of bacterial suspension containing 10^5^ CFU/ml strain IFM 10152 was mixed with 900 μL of *N. farcinica*-negative sputum to form simulated *N. farcinica*-positive sputum.

DNA extraction of the samples mentioned above was performed using DNA purification kits after digestion by 4% sodium hydroxide solution as a previous report described (Qiu et al., 2019). A positive control, a negative control and a blank control were used in each test. The *N. farcinica* CRISPR-CLA assay and the qPCR method were performed using the same DNA templates extracted from clinical samples.

Approval for *N. farcinica* CRISPR-CLA assay validation on clinical samples was provided by the Research Ethics Committee of National Institute for Communicable Disease Control and Prevention, Chinese Center for Disease Control and Prevention (no. ICDC-2021004).

## 3 Results

### 3.1 CRISPR-CLA Design

The CRISPR-CLA design and reaction principle are shown in **Figure 1**. In the CRISPR-CLA system, the forward inner primer (FIP) was modified in the linker region with a PAM site (TTTA) of Cas12a. Thus, the FIP primer of the CRISPR-CLA assay consisted of three regions, including the F1c region (5’-end, complementary to the F1 region), the inserted PAM site (linker, target sequence-independent), and the F2 region (3’-end). As a result, our CRISPR-CLA assay can detect any target sequence even as these sequences did not contain suitable PAM sites. After LAMP preamplification, the amplicons contained the newly acquired PAM site derived from the engineered FIP primer, which can be used for guiding the Cas12a/crRNA complex to recognize and bind the target sequence. If the target sequences were recognized, Cas12a was activated, the *trans-*cleavage activity of which was triggered, resulting in cleavage of the reporter molecule (single-strand DNA, ssDNA). The fluorescence signal was released and captured by a real-time fluorescence instrument. Alternatively, the CRISPR-CLA results could be read out on a lateral flow biosensor within 2 min (**Figures 1A**, **2**). Therefore, the whole reaction of the CRISPR-CLA assay can be completed within 70 min, including rapid DNA extraction (20 min), LAMP preamplification (40 min), CRISPR-based detection (8 min) and readout (fluorescence, real-time; lateral-flow, < 2 min).

### 3.2 Visualization of CRISPR-CLA Results on LFB

As a relatively convenient method, the CRISPR-CLA results could also be visualized on LFB after being reacted in an isothermal metal or water bath. The details about the principle of results readout on LFB are shown in **Figure 2**. After LAMP preamplification, the cleavage reaction was manipulated at 37°C. Then, 8 μL of reaction products was loaded onto the sample pad of the LFB followed by two to three drops running buffer. The running buffer moved along the nitrocellulose membrane through capillary action and rehydrated the immobilized reagent (GNPs-SA) on the conjugate pad. For a negative reaction, the ssDNA was not cleaved, and all the FAM groups were captured by the anti-FAM dispensed on the control line while the biotin groups bound GNPs-SA. Thus, only the control line showed a red band in the negative result. For a positive reaction, the ssDNA was cleaved by the activated Cas12a, and the FAM and the biotin were separated. The biotin-BSA dispensed on the test line captured parted biotin/GNPs-SA complex, while the anti-FAM captured parted FAM groups and noncleaved ssDNA. Both the control line and the test line showed red bands in the positive result.

### 3.3 *N. farcinica* CRISPR-CLA Assay

In this study, the CRISPR-CLA platform was employed to diagnose nocardiosis caused by *N. farcinica* rapidly and accurately (named *N. farcinica* CRISPR-CLA assay). A LAMP primer set (including six primers targeting eight areas) was designed targeting the *pbr1* gene, which was highly specific and conserved in *N. farcinica* and was reported first in this study. To meet the requirement of CRISPR-CLA detection, the FIP primer was modified with a PAM site TTTA at the linker region (**Figure 1** and **Table S1**).

To confirm the effectiveness of the LAMP primers, LAMP amplification using the *N. farcinica* reference strain IFM 10152 was conducted. A negative control (DW) was used. A real-time turbidimeter (Loopamp, LA-320c) was employed to monitor the amplicons. Our results showed that the engineered primers were able to amplify the target sequence within 40 min (**Figure S2A**). Then, CRISPR-based detection was performed with LAMP products. A blank control (DW) was tested simultaneously by the real-time fluorescence reader to verify the validity of the CRISPR-based reaction system. We found that the fluorescence signal of the positive reaction was strongly enhanced within 8 minutes, while those of the negative control and the blank control did not raise significantly (**Figure S2B**). Visualization of *N. farcinica* CRISPR-CLA results on LFB was also carried out. The CRISPR-based reaction systems with positive and negative LAMP products were incubated at 37°C for 8 min using a metal bath (Thermo Scientific, USA). After that, the result reading on LFB was performed according to the CRISPR-CLA workflow. As expected, red bands occurred on both the control line and test line for the positive reaction, while a red band only appeared on the control line for the negative reaction (**Figure S2C**). In particular, the whole process of the *N. farcinica* CRISPR-CLA assay can be completed within 70 min.

### 3.4 Optimal Reaction Conditions for *N. farcinica* CRISPR-CLA Assay

#### 3.4.1 Optimal crRNA Screening

**Figure 3:**
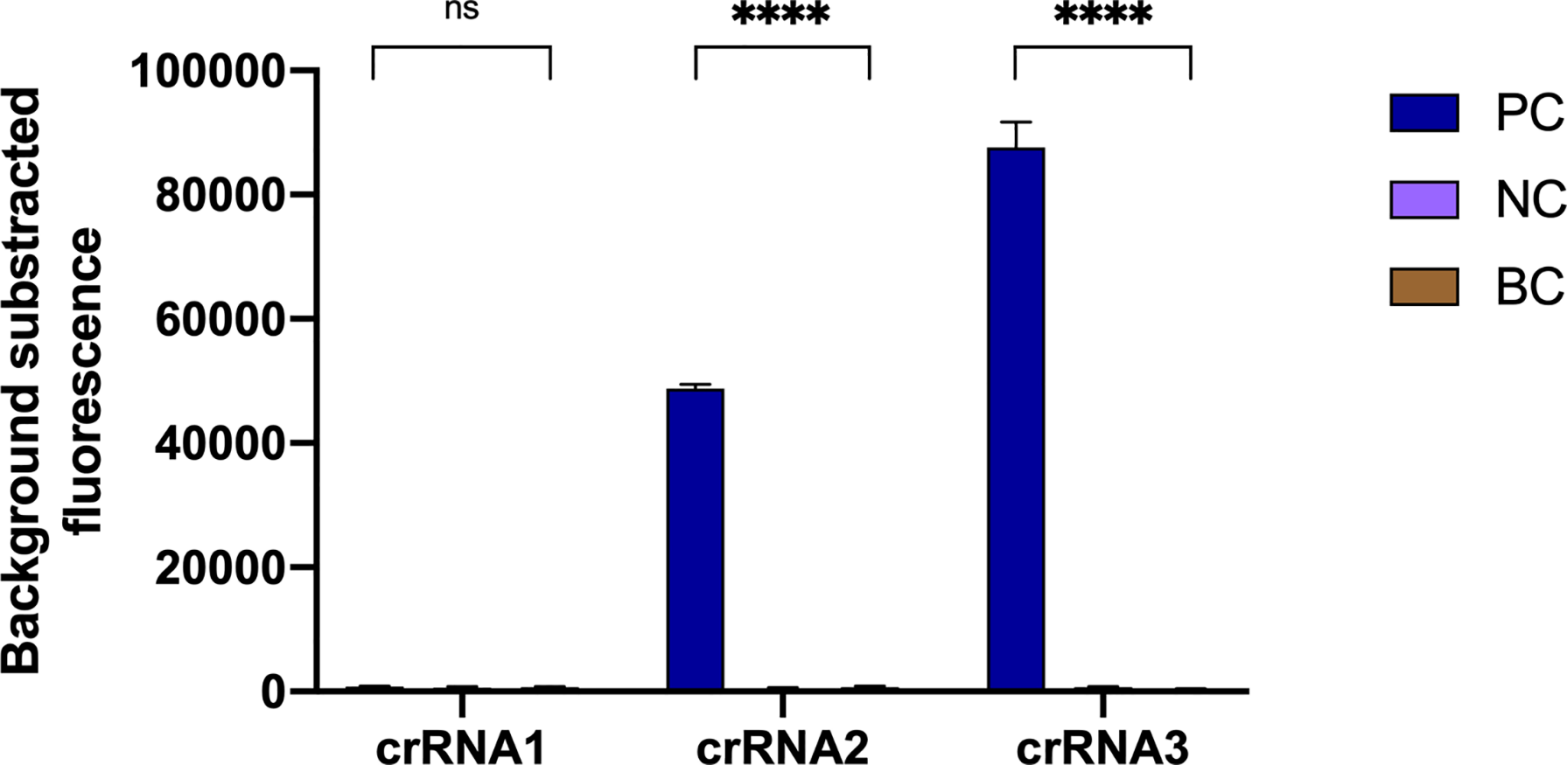
Optimal crRNA screening for the *N. farcinica* CRISPR-CLA assay. The crRNA3 was screened out, because of the highest fluorescence value. PC, positive control; NC, negative control; BC, blank control. (n = 3 technical replicates, two-tailed Student t-test; ns, no significance; ****p < 0.0001; bars represent mean ± s.e.m.).

According to the amplification site, we designed three crRNAs with different lengths (**Table S1**, crRNA1, 39 nt; crRNA2, 41 nt; crRNA3, 43 nt), and then the effectiveness of the crRNAs was compared. Negative controls and blank controls were used. As shown in **Figure 3**, crRNA3 exhibited the highest fluorescence value compared with other crRNAs, while the fluorescence signal of crRNA1 did not obviously increase. Therefore, crRNA3 (43 nt) was considered the most effective crRNA. Then, the *in vitro* cleavage assay with Cas12a and crRNA3 was performed. The result indicated the crRNA can specifically and efficiently cleave the target (**Figure S3**).

#### 3.4.2 Optimal ssDNA Probe and Work Concentration Screening

**Figure 4:**
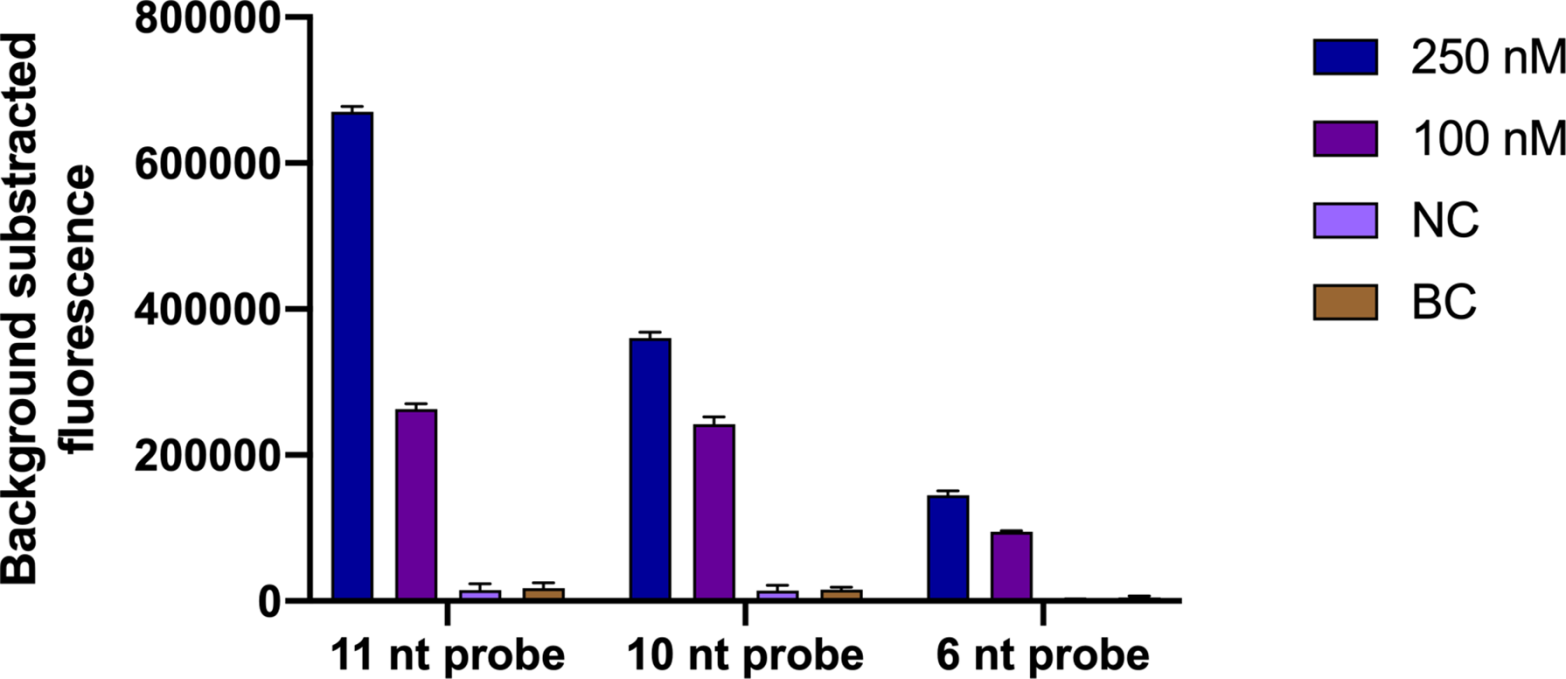
Optimal ssDNA probe and work concentration screening for the *N. farcinica* CRISPR-CLA assay. The 11 nt ssDNA probe with 250 nM work concentration was screened out, because of the highest fluorescence value. nt, nucleotide; NC, negative control; BC, blank control. (n = 3 technical replicates; bars represent mean ± s.e.m.).

According to the principle of Cas12a *trans*-cleavage (Yamano et al., 2017; Chen et al., 2018), three ssDNA probes with different lengths (**Table S1**, 6 nt, 10 nt and 11 nt) were designed. Three probes with two working concentrations were tested in this assay. A negative control and a blank control were used. As shown in **Figure 4**, the fluorescence intensity increased along with the length and working concentration of the ssDNA probe. As a result, the 11 nt probe with a 250 nM concentration hit the highest fluorescence value.

#### 3.4.3 Optimal Reaction Temperature of LAMP Preamplification

To confirm the optimal amplification temperature of LAMP preamplification, isothermal amplification of *pbr1* was executed using *N. farcinica* IFM 10152 (100 pg of genomic DNA per reaction). The reactions were performed at 59-70°C with 1°C increment, and the amplification products were monitored by a real-time turbidimeter. According to the data, 70°C was considered the optimal temperature for LAMP preamplification of *pbr1* due to the fastest amplification and the high-level products (**Figure S4**).

### 3.5 Specificity of the *N. farcinica* CRISPR-CLA Assay

Specificity of *N. farcinica* CRISPR-CLA assay was determined using 132 isolates, including bacteria and fungi (**Table S2**). *N. farcinica* IFM 10152 was used as the positive control. A negative control and a blank control were used. The generated data showed that all positive results were obtained from *N. farcinica* strains, whereas negative results were obtained from non-*N. farcinica* strains, which indicated that the *N. farcinica* CRISPR-CLA assay is highly specific with no cross-reaction for non-*N. farcinica* agents. It is worth noting that the results of the lateral-flow assay were in complete accordance with those of the fluorescence assay.

### 3.6 Sensitivity of the *N. farcinica* CRISPR-CLA Assay

**Figure 5:**
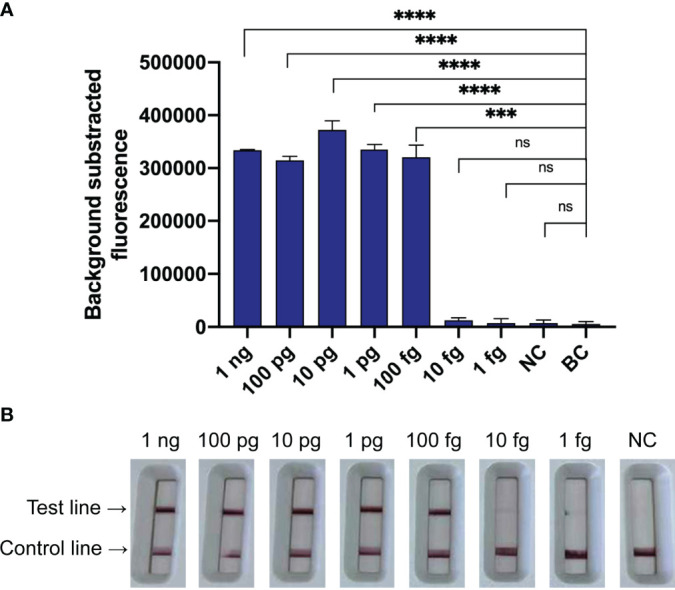
Sensitivity of the *N. farcinica* CRISPR-CLA assay. **(A)** Sensitivity of the fluorescence *N. farcinica* CRISPR-CLA assay. One hundred fg dsDNA was determined as the LoD of the fluorescence *N. farcinica* CRISPR-CLA assay. (n = 3 technical replicates, two-tailed Student t-test; ns, no significance; ***p < 0.001; ****p < 0.0001; bars represent mean ± s.e.m.) **(B)** Sensitivity of the lateral-flow *N. farcinica* CRISPR-CLA assay. One hundred fg dsDNA was determined as the LoD of the lateral-flow *N. farcinica* CRISPR-CLA assay. NC, negative control. BC, blank control.

The LoD of the *N. farcinica* CRISPR-CLA assay for pure culture was 100 fg of dsDNA per reaction (equivalent to 10^-21^ M) using seven serial dilutions (1 ng to 1 fg with 10-fold interval) of genomic DNA extracted from *N. farcinica* IFM 10152 (**Figure 5**). The fluorescence *N. farcinica* CRISPR-CLA assay and the lateral-flow *N. farcinica* CRISPR-CLA assay were performed independently, and similar results of the two assays were acquired. In the result readout of the lateral-flow *N. farcinica* CRISPR-CLA assay, the visual bands, indicating absence or presence of target sequences, were produced by the biosensors, and the results were interpreted easily at all detectable levels (**Figure 5B**). The sensitivity of the real-time qPCR detection was tested using same DNA templates with CRISPR-CLA assay and the LoD of qPCR was also 100 fg of dsDNA per reaction (**Figure S5**).

### 3.7 Feasibility of the *N. farcinica* CRISPR-CLA Assay Using Clinical Samples

**Figure 6:**
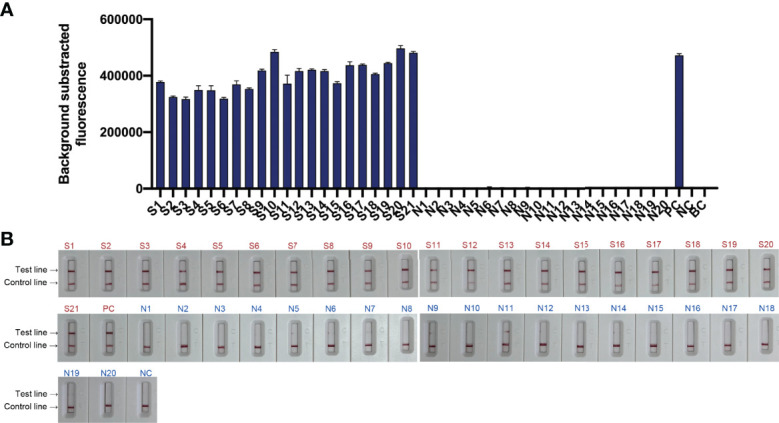
Feasibility of the *N. farcinica* CRISPR-CLA assay using clinical samples. **(A)** The fluorescence *N. farcinica* CRISPR-CLA assay results. Sample S1 to S21 showed positive results and sample N1 to N20 showed negative. (n = 3 technical replicates; bars represent mean ± s.e.m.) **(B)** The lateral-flow *N. farcinica* CRISPR-CLA assay results. Sample S1 to S21 showed positive results and sample N1 to N20 showed negative. S1-S20, simulated positive samples; S21, positive sample; N1-N20, negative samples; PC, positive control; NC, negative control; BC, blank control.

To evaluate the feasibility of *N. farcinica* CRISPR-CLA assay to clinical samples, the nucleic acids from 41 sputum specimens, including twenty simulated *N. farcinica*-positive samples (S1-S20), one *N. farcinica*-positive sample from a nocardiosis patient (S21), and twenty *N. farcinica*-negative samples (N1-N20) were detected by fluorescence and lateral-flow assays. As expected, 21 positive results were obtained from S1 to S21, whereas 20 negative results were from N1 to N20 (**Figure 6**). The results initially suggested that the *N. farcinica* CRISPR-CLA assay could be a suitable and valuable detection scheme for *N. farcinica* discovery in the clinic.

A real-time qPCR detection assay was also used to detect the 41 samples. As shown in **Figure S6**, 15.0% (3/20) of *N. farcinica*-negative samples showed false-positive results.

## 4 Discussion

Under the COVID-19 pandemic background, nucleic acid detection has become the gold standard to rapidly diagnose the infectious disease. A rapid, low cost, reliable nucleic acid detection platform will be the key to control next potential pandemic. In the present study, a CRISPR-based diagnostic platform, termed CRISPR-CLA, which integrated loop-mediated isothermal amplification (LAMP) with CRISPR/Cas12a-based detection, was developed. Two assays, by a fluorescence reader and by a lateral-flow biosensor, could be used in different situations. To demonstrate its validity, CRISPR-CLA was applied to detect *N. farcinica*, a long-term neglected pathogen, in pure cultures and clinical samples.

Recently, several CRISPR-mediated detection methods have been established to diagnose diseases at the molecular level (Gootenberg et al., 2017; Chen et al., 2018; Chertow, 2018; Li et al., 2018a; Li et al., 2018b; Myhrvold et al., 2018; Ai et al., 2019; Liang et al., 2019; Teng et al., 2019; Wang et al., 2021). These nucleic acid detection platforms exhibited high sensitivity and extreme specificity in molecular diagnosis. For these CRISPR-based nucleic acid detection techniques, a proper preamplification step is usually vital to raise the sensitivity of detection. PCR and some isothermal amplification methods, such as recombinase polymerase amplification (RPA) and multiple cross displacement amplification (MCDA), have been employed to establish CRISPR-based detection platforms (Ai et al., 2019; Li et al., 2021; Zhu et al., 2021). In these assays, infectious diseases with various pathogens, including bacteria, viruses and fungi, can be diagnosed accurately.

Despite exhibiting high sensitivity and ultraspecificity, a CRISPR-based detection platform may be restricted in practical application due to lack of a suitable PAM site in the target sequence. In the regular LAMP reaction, the FIP consisted of the F1c region and the F2 region (Notomi et al., 2000). In the CRISPR-CLA method, a PAM site (TTTA) of the Cas12a effector was inserted between the F1c region and the F2 region to form the engineered FIP (**Figure 1B**, 1C). Although the amplification efficiency may be compromised due to the inserted PAM sequence, our CRISPR-CLA eliminated the PAM site limitation by the engineered amplification primer. Along with the LAMP reaction using the engineered FIP, the PAM sites and the crRNA binding sites increased exponentially in certain LAMP products (**Figure 1C**). Thus, CRISPR-CLA can be applied to detect any sequence that meets the requirement of primer design of LAMP, even if the sequences lack suitable PAM sites of Cas12a. In addition, the CRISPR-CLA assay is economic. Many commercial kits (such as DNA isothermal amplification kits and Eiken Loopamp Kits) can be used to perform LAMP preamplification and Lba Cas12a is not expensive (250 USD/2,000 pmol). Therefore, each reaction of CRISPR-CLA assay costs <8 USD.

In this study, the *pbr1* gene was first reported as the highly species-specific target of *N. farcinica*, which opens the door to research the molecular mechanism and the development of other detection assays of *N. farcinica* in the future. The *pbr1* gene, encoding a PQQ-binding-like beta-propeller repeat protein, is highly conserved in *N. farcinica* and is nonexistent in other non-*N. farcinica*, which indicates that this gene is a favorable target for *N. farcinica* detection. There is large potential for mining the gene function and applying *pbr1*.

*N. farcinica* CRISPR-CLA assay targeting the *pbr1* gene was developed and exhibited extreme specificity. At the isothermal preamplification stage, six LAMP primers recognizing eight regions of the *pbr1* gene ensured high specificity for *N. farcinica* detection. LAMP has shown specificity in previous reports (Notomi et al., 2000; Dong et al., 2015; Yan et al., 2020); random cross-amplification caused by primers, however, may occur after long-term reactions (Ding et al., 2020; Rolando et al., 2020; Tian et al., 2020). In the *N. farcinica* CRISPR-CLA assay, the crRNA recognized and bound with the target sites in the preamplicons under the guidance of the PAM sites, which further improved the specificity of the detection (single-nucleotide target specificity). Our data also demonstrated that no positive results were obtained from non-*N. farcinica* templates (**Table S2**). Therefore, the CRISPR-CLA assay was able to accurately detect *N. farcinica*. The LoD of the *N. farcinica* CRISPR-CLA assay was 100 fg dsDNA per reaction, which indicated the assay had good sensitivity. Moreover, the LoDs of the fluorescence *N. farcinica* CRISPR-CLA assay and the lateral-flow *N. farcinica* CRISPR-CLA assay were concordant (**Figures 5**, **6**), which indicated that the LFB can be a smart alternative to a fluorescence reader and is especially suitable for point-of-care testing and on-site diagnosis.

The *N. farcinica* CRISPR-CLA assay was initially validated using the extracted DNA from clinical samples. In total, 41 sputum samples were examined using the *N. farcinica* CRISPR-CLA assay and a qPCR method. According to our data, the sensitivity of *N. farcinica* CRISPR-CLA assay was similar with that of the qPCR method. However, three *N. farcinica*-negative samples exhibited false-positive results by the qPCR method (**Figure 6**, **Figure S6** and **Table S3**). Our results suggested that the *N. farcinica* CRISPR-CLA assay was more specific than the qPCR method and was a reliable method for diagnosis of *N. farcinica* infection. The lateral-flow *N. farcinica* CRISPR-CLA assay is more suitable for POC or on-site testing than the qPCR method. For negative results, sometimes, a faint signal may occur at the test line, which results from the noncleaved ssDNA probes not being adequately captured by anti-FAM at the control line, but it was significantly fainter than a positive signal, which will not influence the result readout (**Table S4**), and a repeat using another LFB can also solve the confusion easily. Therefore, the lateral-flow *N. farcinica* CRISPR-CLA assay is usually recommended because the LFB is portable, easy to use, and equipment independent, especially for POC and on-site testing.

As in a previous study (Qiu et al., 2022), a CRISPR-CPA (CRISPR/Cas12a-based detection combined with PCR amplification) nucleic acid detection platform was developed to diagnose nocardiosis caused by *N. farcinica*. However, special equipment and much more reaction time are needed by PCR amplification, which is a limitation for point-of-care (POC) and on-site tests. In this report, a steady isothermal amplification assay LAMP was used as the amplification agent because of its rapidity, easy-to-use and low cost. In spite of the simplicity of PCR primer design, LAMP has particular advantages, especially in on-site work. For example, precision instruments are not necessary for the CRISPR-CLA platform, and an isothermal heating block and portable LFBs are enough to access exact results, which is very suitable for POC and field work. Although both CRISPR-CPA and CRISPR-CLA showed ultraspecificity, the CRISPR-CLA assay showed tenfold sensitivity than CRISPR-CPA (100 fg for CRISPR-CLA vs. 1 pg for CRISPR-CPA). Without the thermocycling instrument requirement, the CRISPR-CLA assay saved significant turnaround time. The completed detection process, including DNA extraction (20 min), LAMP preamplification (40 min), CRISPR-based detection (8 min) and readout of the results (within 2 min), was completed in 70 min (**Figure 1A**). Therefore, a shorter detection time (70 min for CRISPR-CLA vs. 90 min for CRISPR-CPA) is another advantage of CRISPR-CLA compared to CRISPR-CPA. Considering the advantages, the CRISPR-CLA assay can be a potential diagnostic tool in on-site work.

## 5 Conclusion

In this report, a CRISPR/Cas12a-based diagnostic platform, termed CRISPR-CLA, was developed and applied to *N. farcinica* detection. Moreover, the *pbr1* gene was first reported as a species-specific gene of *N. farcinica*. The results of the *N. farcinica* CRISPR-CLA assay can be read out by a portable LFB, and the whole process can be finished within 70 min. Our results indicated that the *N. farcinica* CRISPR-CLA assay was rapid, low-cost, and ultraspecific, which provided a valuable tool for accurate diagnosis of *N. farcinica* infection, especially for POC and on-site testing.

## Data Availability Statement

The original contributions presented in the study are included in the article/**Supplementary Material**. Further inquiries can be directed to the corresponding author.

## Ethics Statement

The studies involving human participants were reviewed and approved by Research Ethics Committee of National Institute for Communicable Disease Control and Prevention, Chinese Center for Disease Control and Prevention. The patients/participants provided their written informed consent to participate in this study.

## Author Contributions

XQ designed and performed the experiments, analyzed the data, showed the visualization and wrote the original draft. SX selected the target gene. XL performed part of the experiments and showed part of the visualization. HR provided the resources. ZL proposed the concept, provided financial and administered the project. All authors contributed to the article and approved the submitted version.

## Funding

This work was supported by the National Key Research and Development Program of China [grant numbers 2019YFC1200700, 2019YFC1200601-6, 2021YFC2401000, 2021YFC2301105] and the National Natural Science Foundation of China [grant number 82073624].

## Conflict of Interest

The authors declare that the research was conducted in the absence of any commercial or financial relationships that could be construed as a potential conflict of interest.

## Publisher’s Note

All claims expressed in this article are solely those of the authors and do not necessarily represent those of their affiliated organizations, or those of the publisher, the editors and the reviewers. Any product that may be evaluated in this article, or claim that may be made by its manufacturer, is not guaranteed or endorsed by the publisher.
